# Eczema in Infants

**Published:** 1899-02

**Authors:** 


### ECZEMA IN INFANTS.

At a meeting of the British Medical Association recently held in Edinburgh the discussion as to the origin of eczema was deeply interesting.

Mr. Malcolm Morris upheld to a great extent Unna's view that the disease is of parasitic origin, going so far as to declare that he was of the opinion that parasites were capable of producing eczema even if it were not actually a parasitic disease. Dr. Talcott Fox said that since the promulgation of Unna's ideas on eczema he had determined to study again the condition known as eczema, unbiased, as far as possible, by the learnings of his early training.

In offering a few observations on the disease in children, he said he did so because in them the problem was, in a measure, free from some of the complications met with in later life. In infants and children he saw two phases of eczema. The first was the state commonly known as eczema, which began most frequently in infancy on the scalp and descended thence over the face, to cover, in some instances, the greater part of the surface. The second phase consisted of dry, reddish areas, with scanty, adherent scales, separating along the lines of flexion; but these patches might also, when irritated, take on a vesicular form, indistinguishable from the first phase. This was also a descending inflammation, beginning on the face or scalp, as a rule, which he used to call seborrhea; but that was no doubt a bad term. This was the common affection described by Savell as epidemic among children. These phases ran into one another so much that he had constantly the greatest difficulty in separating them. In the next place he could find no cause whatever in the majority of children, for the patients were apparently in excellent health. He did not deny the influence of constitutional disturbance such as rickets or gastro-intestinal trouble in increasing the irritation and intensifying the inflammation, but the phases of eczema seemed to be essentially of local origin. It was possible that the future might disclose varieties caused by various parasites. The theory of parasites as a cause of eczema is rapidly gaining ground. Professor Lassar, of Berlin, scouts the idea of irritation from internal sources being a cause of eczema and points out how favorable are the skin changes in eczema to the growth of saprophytic and pathogenic micro-organisms.—*Pediatrics.*

